# Ultrasound of horizontal instability of the acromioclavicular joint: A simple and reliable test based on a cadaveric study

**DOI:** 10.1007/s00508-018-1433-x

**Published:** 2019-01-07

**Authors:** Gerhard Martin Hobusch, Kilian Fellinger, Tobias Schoster, Susanna Lang, Reinhard Windhager, Manuel Sabeti-Aschraf

**Affiliations:** 1 Department of Orthopaedic Surgery, Medical University of Vienna, Waehringer Guertel 18–20, 1090 Vienna, Austria; 2 Department of Pathology, Medical University of Vienna, Vienna, Austria

**Keywords:** Horizontal instability, Rockwood classification, Shoulder sonography, Acromioclavicular joint

## Abstract

**Background:**

Horizontal instability influences the clinical outcome after acromioclavicular joint (ACJ) injuries and in joint degeneration. A standardized, dynamic examination of the horizontal instability has not been described before. This current study presents a sonographic method to analyze the dynamics between the clavicle and acromion in the horizontal plane.

**Methods:**

The horizontal joint play, the ACJ space and the offset between clavicle and acromion were sonographically assessed by a 45° ventrally angulated longitudinal section. A total of four investigators with different experience in the field of musculoskeletal ultrasound examining 20 ACJs in 10 human cadavers. Measurements in the absence of any pressure and under standardized anterior and posterior pressure onto the clavicle were carried out in different ligament status intact acromioclavicular (AC) and coracoclavicular (CC) ligaments as well as in a Rockwood (RW) II and III model. A two-sided t‑test was used to examine the differences between joint positions and ligament status.

**Results:**

The horizontal joint play was 1.3 ± 0.9 mm with intact ligaments, 1.4 ± 1.2 mm by transecting the AC ligaments and 1.9 ± 1.3 mm after additionally transecting the CC ligament. The joint space was 3.3 ± 1.1 mm with intact ligaments, 4.1 ± 1.8 mm in an iatrogenic RW 2 injury, and 5.3 ± 3.3 mm in an iatrogenic RW 3 injury. Manipulating the clavicle by applying anterior or posterior pressure did not change the difference within one injury pattern. Interobserver reliability was 83.9%.

**Conclusion:**

Apart from evaluating the ligaments and the joint capsule, measurement of the dynamic horizontal instability is possible in a human cadaver model. The ultrasound-based measurement of horizontal instability dynamics avoids radiation exposure, is readily available and cost-efficient.

## Introduction

Stability of the acromioclavicular (AC) joint is provided by the capsular ligaments and the coracoclavicular (CC) ligaments and is subdivided in vertical and horizontal stability [4, 8]. Horizontal instability occurs after a horizontal blow to the clavicle in a fixed scapula and acromion. It is clinically relevant as it has been described to be associated with increased pain and to occur after Rockwood (RW) type 2 injuries or after distal clavicle resection [2, 9, 11, 13]. Varying forms of horizontal instability combined with vertical instability of the ACJ may be the reason for a lack of uniform treatment in RW type 3 injuries [16]. There is a trend in surgery towards anatomically restoring the ACJ, i.e. to treat vertical as well as horizontal instability to obtain a better functional outcome [10].

Ultrasound (US) is a valuable imaging technique for displaying abnormalities of the ACJ with dynamic evaluation in the form of the cross-arm maneuvre being suitable for the detection of mild or moderate sprains in AC separation [12, 15]. Although the direct sonographic visualization of the ACJ is a very sensitive imaging modality considering vertical instability, a standardized dynamic examination of the horizontal instability using ultrasound has not been described [5–7, 12, 14].

The aim of this study was to study the precision of US in diagnosing horizontal instability in an experimental human cadaver model.

## Material and methods

This study was planned and carried out as a prospective randomized cadaver trial, after receiving a positive ethics commission vote (EK Nr. 1764/2014). The study was carried out on 10 frozen and thawed corpses with the following inclusion criteria: age between 18 and 100 years, no infections, no recent trauma in both shoulders, no known paralysis in both shoulders, no obvious deformity in the area of the clavicle or acromion, which was reported by chart reviews. The mean age was 74.2 years, the mean weight was 71.5 kg and the mean height was 1.67 m. Therefore, the mean body mass index (BMI) was 25.5 kg/m^2^, 6 (60%) of the corpses were female and 4 (40%) male.

The subjective mobility of the whole frozen and thawed corpses, depending on temperature and time of death, was in proportion flexible to medium hard to stiff = 4:3:3 (Fig. 5).

### Sonographic method

**Fig. 1:**
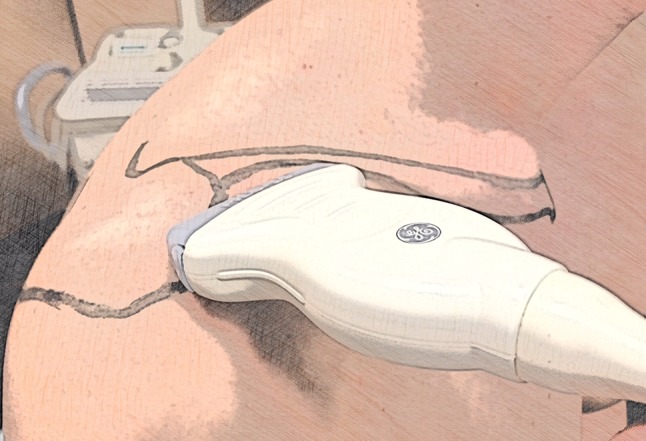
Position of the scan to monitor horizontal instability of the ACJ

**Fig. 2:**
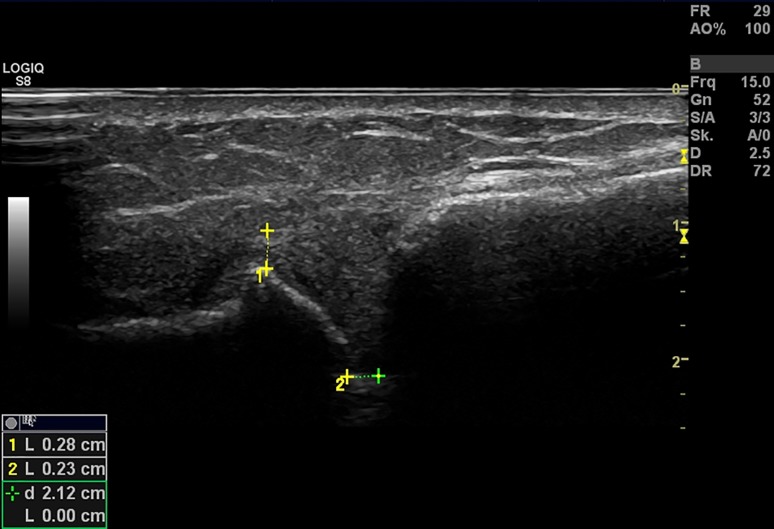
A fixed US image was made from 45° transverse plane, on which the joint space, a zone of mean echogenicity, as well as the ACJ offset were both measured as a distance

The distance between the ACJ and sonic head was measured without any manipulation of each joint. A 45° ventrally angulated longitudinal section plane was used to display the acromion, clavicle and the ACJ space (Fig. 1). The joint space as well as the ACJ offset were both measured as distances from this plane (Fig. 2). Ultrasound was carried out using a GE Healthcare (GE Healthcare, Chicago, IL, USA), Logiq S8, software version Logiq S8: R1.5.4, linear probe: GE Healthcare ML6-15, 15.0 Mhz (xx). The shoulders of each corpse were measured by two orthopedic consultants and two students. Without having experience in the field of musculoskeletal ultrasound, the students had a short theoretical and practical introduction into the US technique and interpretation and were guided how to use the sonic head and how to physically measure distances. Images were taken to determine the horizontal instability by applying constant manual anterior and posterior pressure onto the clavicles. Prior to the study anterior and posterior pressure onto the clavicle was repeatedly measured by pressure sensors (Pressure sensor Interlink FSR 400 Multimeter: Voltcraft, VC 130) until the desired resistance of 100 kgohm was reached straightaway and not exceeded by every investigator. In order to avoid interference with the study set-up pressure sensors were not used during measurement.

### Surgical model

**Fig. 3:**
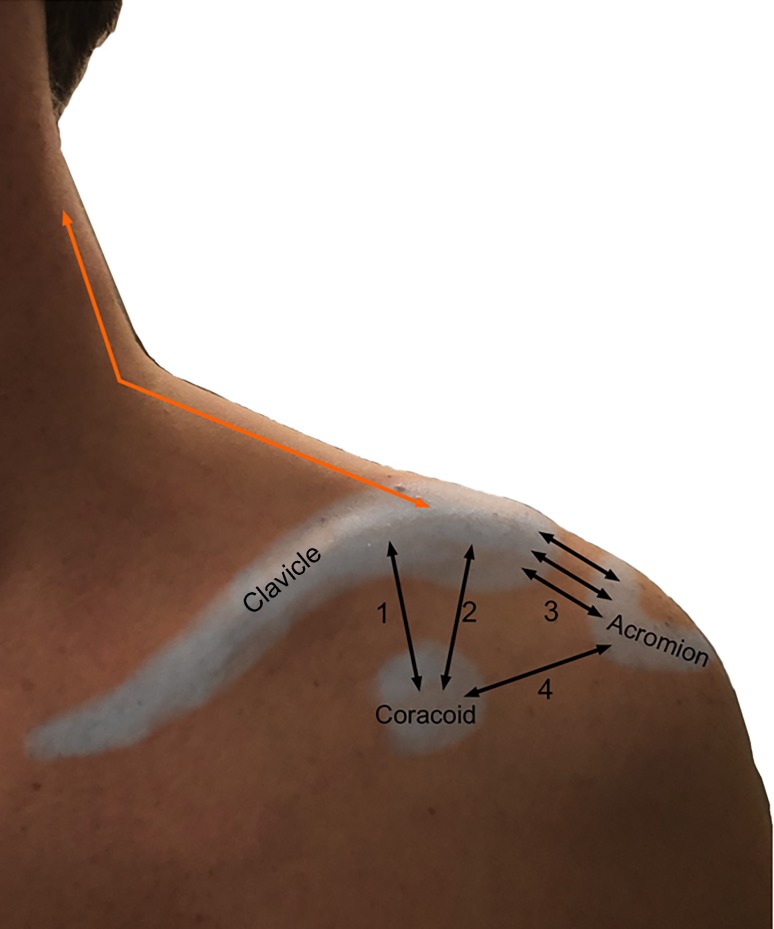
Schematic representation of the ligaments involved in the biomechanics of the ACJ (*1* conoid ligament, *2* trapezoid ligament, *1* *+* *2* coracoclavicular ligament, *3* acromioclavicular ligament, *4* coracoacromial ligament, *red* trapezius muscle)

**Fig. 4:**
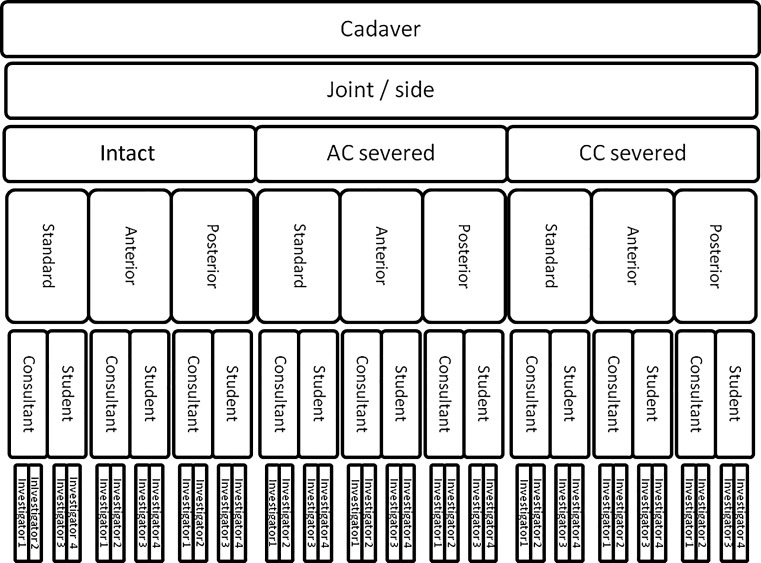
Flowchart showing how measurements for each cadaver were organized

After imaging of the same ACJ in three different pressure modes (without pressure, anterior and posterior pressure) the acromioclavicular ligament (ACL) was sharply transected with a scalpel under sonographic control, making a 1 cm ventral skin incision above the ACJ. After simulating this RW 2 injury the measurement of the horizontal instability imaging was done as mentioned (without pressure, anterior and posterior pressure). Following this the coracoclavicular ligaments (CCL) were transected. Thereby the known skin incision was used again to subsequently transected both the trapezoid ligament and conoid ligament by withdrawing a backwardly bladed knife alongside the clavicle from medial to lateral thereby reaching the injury pattern of RW 3 (Fig. 3). Once again imaging procedures were carried out as mentioned (without pressure, anterior and posterior pressure). This way nine distances regarding horizontal instability and nine distances regarding information about the joint space were measured in each joint by all four examiners (Fig. 4).

### Data management and evaluation

**Fig. 5:**
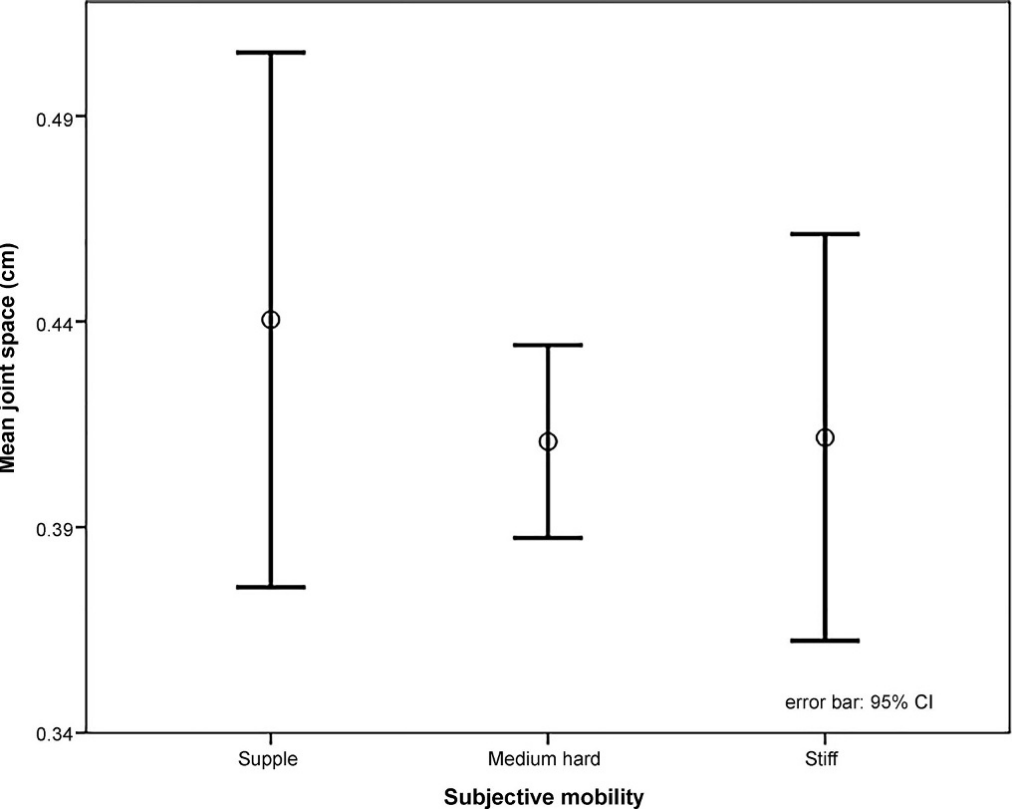
Subjective mobility of the corpses depending on temperature and time of death was in the proportions supple to medium hard to stiff = 4:3:3; Difference was measured in the standard value (as to middle hard and stiff)

All measurements were immediately transferred onto case reports forms. The subjective mobility of the corpses was assessed 1 (Fig. 5) as they were not assessed before imaging for fear of tissue damage. The investigators carried out the measurements in a random order.

Due to the relative joint play the absolute value was formed as a difference between the anterior and posterior horizontal joint play.

$$\text{absolute}=\text{anterior}\,-\,\text{posterior}$$

This value was used for statistical evaluation. Mean absolute values were arranged in the following groups and compared by two-sided *t *-tests, as well as correlation analysis according to Pearson. Groups were formed according to the state of the ligament (intact, AC transected, CC transected), to left and right side, to side and state of ligament, to examiner as well as to training and to consultant or student, compared with each other and cross-analyzed. The two-sided *t *-test was also used to examine the differences between joint positions (without pressure, with pressure to the anterior side or pressure to the posterior side) and ligament status (intact, AC transected, CC transected). Then correlation analysis between ligament status and horizontal relocatability was examined.

## Results

**Fig. 6:**
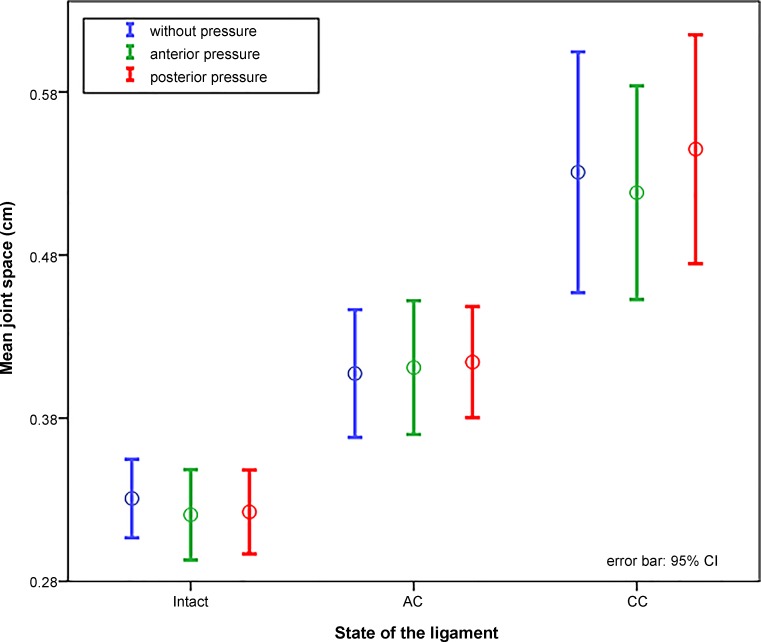
Mean absolute values of all investigations, by all 4 investigators were arranged within groups according to the 3 states of the ligament (intact, AC transsected, CC transsected) and 3 joint positions (without pressure, with pressure to the anterior side or pressure to the posterior side) and compared by two sided *t*-tests

The mean distance of the ACJs to the sonic head was 10.1 ± 0.2 mm. The horizontal joint play was 1.3 ± 0.9 mm with intact ligaments, 1.4 ± 1.2 mm by transecting the AC ligaments and 1.9 ± 1.3 mm after additionally transecting the CC ligament (Fig. 6). This examination was regardless of examiner (training and individual) and patient (side, mobility of the corpse, distance of the ACJ to the sonic head). No changes were found in the chronological sequence of measurements.

**Fig. 7:**
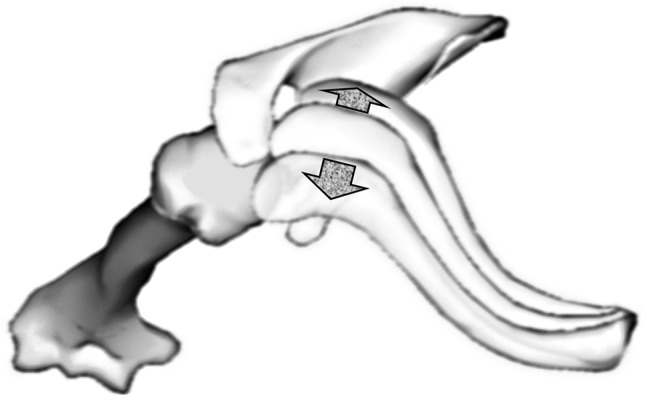
View of a scapula from cranial. Anterior and posterior pressure show the directions of horizontal instability. (BodyParts3D, [http://lifesciencedb.jp/bp3d/] © The Database Center for Life Science licensed under CC Attribution-Share Alike 2.1 Japan, Japan)

The joint space was 3.3 ± 1.1 mm with intact ligaments, 4.1 ± 1.8 mm in an iatrogenic RW 2 injury, and 5.3 ± 3.3 mm in an iatrogenic RW 3 injury. Manipulating the clavicle by applying anterior or posterior pressure did not change the difference within one injury pattern (Fig. 7; [17]). There was no difference in the joint space of different sides. These examinations can therefore differentiate the joint space between a healthy joint and an ACJ RW 2 or RW 3 injury regardless of patient and investigator.

The offset between clavicle and acromion in the horizontal plane without manipulation was taken as a reference value, as neither the condition of the ligament nor the side of the patient had any effect. In flexible corpses there was a significantly larger offset (2 ± 1.2 mm) as with medium hard (1.7 ± 1.1 mm) or stiff (1.6 ± 1.1 mm). There was no significant difference between consultant and student measurements. There were no differences in 83% of the offset values between different investigators. Comparing the ACJs of the different sides showed a larger horizontal offset of the clavicle to acromion of the right shoulder (1.9 ± 1.3 mm) compared to the left shoulder (1.7 ± 1.0 mm), whereas sides correlated with each another. The horizontal joint play was the same on the right and left side and there was no significant correlation. Interobserver reliability in 5 out of 6 comparisons (83.9%) showed no differences in the joint space as well as in joint play. The different levels of training did not have any influence on the examination of the horizontal offset and horizontal joint play; however, there was a difference when measuring the joint space between orthopedic consultants and students. Values and standard deviations (SD) regarding the analysis of the measurements of all four investigators over time were constant.

## Discussion

In this study an US method was developed and tested for detecting horizontal instability of the ACJ with high precision. As expected, the measured horizontal instability increased when cutting the ligaments. These results correspond to the results of Dawson et al. who investigated the biomechanics and the instability by transecting AC and CC ligaments in human cadavers [3].

With US measurement of the joint space width Rockwood types I–III can be differentiated on static images, i.e. independent of any pressure applied to the clavicle. As the differences between the dissected and the non-dissected contralateral ACJ were significant, US measurements of the contralateral side may be used for comparison. The thickness of the periarticular soft tissue expressed as the distance between the US probe and ACJ did not influence the measuring accuracy and precision. Although there was a significant difference in the joint space between the measurements of consultants and students, there were no significant differences to be found in a direct comparison of the investigators. Therefore, it could be expected that this method is very safe for an individual investigator even with limited expertise.

The limitation of this study is that in cadaveric specimens the muscle tension is much lower than in living individuals and clinical information, such as left or right dominance of the hand were not available [1].

## Conclusion

Dynamic US is a safe and readily available imaging technique for the precise assessment of the horizonal instability of the ACJ. With static US the joint space width directly correlates with Rockwood stages.

## Abbreviations

ACJ: Acromioclavicular joint
ACL: Acromioclavicular ligament
CCL: Coracoclavicular ligament
RW: Rockwood classification
